# Three-Dimensional Evaluation of Soft Tissue Malar Modifications after Zygomatic Valgization Osteotomy via Geometrical Descriptors

**DOI:** 10.3390/jpm11030205

**Published:** 2021-03-13

**Authors:** Elena Carlotta Olivetti, Federica Marcolin, Sandro Moos, Alberto Ferrando, Enrico Vezzetti, Umberto Autorino, Claudia Borbon, Emanuele Zavattero, Giovanni Gerbino, Guglielmo Ramieri

**Affiliations:** 1 Department of Management and Production Engineering, Politecnico di Torino, Corso Duca degli Abruzzi 24, 10129 Turin, Italy; federica.marcolin@polito.it (F.M.); sandro.moos@polito.it (S.M.); alberto.ferrando@studenti.polito.it (A.F.); enrico.vezzetti@polito.it (E.V.); 2 Department of Surgical Sciences, Division of Maxillofacial Surgery, University of Turin, Città della Salute e della Scienza HOSPITAL, 10129 Turin, Italy; umberto.autorino@unito.it (U.A.); claudia.borbon@unito.it (C.B.); emanuele.zavattero@gmail.com (E.Z.); giovanni.gerbino@unito.it (G.G.); guglielmo.ramieri@unito.it (G.R.)

**Keywords:** 3D face analysis, geometrical descriptors, malar augmentation, zygomatic osteotomy, orthognathic surgery, soft tissue prediction

## Abstract

Patients with severe facial deformities present serious dysfunctionalities along with an unsatisfactory aesthetic facial appearance. Several methods have been proposed to specifically plan the interventions on the patient’s needs, but none of these seem to achieve a sufficient level of accuracy in predicting the resulting facial appearance. In this context, a deep knowledge of what occurs in the face after bony movements in specific surgeries would give the possibility to develop more reliable systems. This study aims to propose a novel 3D approach for the evaluation of soft tissue zygomatic modifications after zygomatic osteotomy; geometrical descriptors usually involved in face analysis tasks, i.e., face recognition and facial expression recognition, are here applied to soft tissue malar region to detect changes in surface shape. As ground truth for zygomatic changes, a zygomatic openness angular measure is adopted. The results show a high sensibility of geometrical descriptors in detecting shape modification of the facial surface, outperforming the results obtained from the angular evaluation.

## 1. Introduction

The last two decades have seen the introduction to medicine of new methodologies founded on 3D technologies enabling a new approach in all fields, from diagnostic to surgery. It has enabled a more accurate process of detection of pathologies as well as a more accurate planning of surgeries. Particularly, the introduction of 3D cone beam computed tomography (3D CBCT) allows to obtain a more precise evaluation of patient conditions with the acquisition of sectional images and to construct three dimensional models helping doctors in better understanding patients’ conditions with lower radiation doses, lower costs, and easier acquisition of images in comparison to traditional computed tomography (CT) and conventional radiography. Concerning the surgical field, maxillofacial and orthognathic surgery has benefitted from the advance in computed tomography technology that enabled systems to support planning and simulation of customized operations [1,2]. The accuracy introduced with 3D CBCT allowed the development of software intended to predict future facial appearance of patients after simulated bony movements [3,4,5,6,7]. Often, these predictions have been judged to be somewhat inaccurate in forecasting soft tissue displacements [8,9,10,11,12]. To better understand how a more reliable prediction tool could be implemented, it is of primary importance to study and quantify the changes occurring in patients who already underwent the surgeries whose effects are to be previewed [13,14]. Thus, focusing on a specific type of surgical operation seems to be a reasonable approach. Several works have been carried out to evaluate facial changes after malar repositioning. Chong Zou at al. evaluated changes of the zygomatic complex to investigate the association between the width of the bony resection and the changes in malar bony parameters, and their effects on facial attractiveness [15]. The identification of reliable measurements considering the malar morphology, summit, and symmetry seemed to significantly help in the postoperative assessment of residual bone asymmetry, as well as in the preoperative planning of zygoma positioning [16]. Changes in visible volume in the malar midfacial area were expected after Le Fort I osteotomy with advancement; the observation of modifications due to this movement introduced an approach to the assessment of normative 3D data of the midfacial region for the soft tissue-to-hard tissue ratio [17]. The volume change of the malar midfacial region was highlighted, with increments and decrements not significantly different from left to right. Moreover, weight changes in patients involved were considered to assess that the volume changes were completely due to malar surgery. The three-dimensional evaluation of malar changes is also a focal point in the assessment of the suitability of surgical techniques in treatment of facial deformities [18]; a subjective and objective evaluation of the pedicled buccal fat pad technique for malar augmentation was carried out by Hernandez-Alfaro et al. considering patients’ pain and satisfaction after surgery as well as the volumetric outcome. The results showed a positive outcome in terms of patients’ satisfaction, with a stable augmentation of the malar volume [19].

Among the surgical techniques proposed to reach a malar augmentation [18,19,20,21,22,23,24,25], one is the malar valgization osteotomy in combination with orthognathic surgery. This surgical approach is performed on patients with maxillary deficiency and an excessive relative mandibular growth; maxilla-mandibular osteotomies address malocclusion and improve the aesthetic of the lower third of the face, the area around the mouth, and the para-latero-nasal area, but do not address flat cheekbones. This causes an unsatisfactory outcome, as in Western societies flat zygoma are considered unattractive [18]. In this sense, an enhancement of the fullness of the zygomatic region leads to a more harmonious facial aspect.

The aim of this study was to propose a novel three-dimensional analysis of the changes occurring in the zygomatic area after this specific type of malar augmentation. Due to their discriminative power in facial analysis applications, i.e., face recognition [26,27] and facial expression recognition [28], geometrical descriptors were involved in this study [29,30]. Particularly, geometrical descriptors from differential geometry were used to highlight malar modifications, while angular measurements have been used as the ground truth of the quantification of these modifications. Despite the low sample size, the results of this exploratory analysis suggest that geometrical descriptors are effective in describing malar modifications.

## 2. Materials and Methods

**Figure 1:**
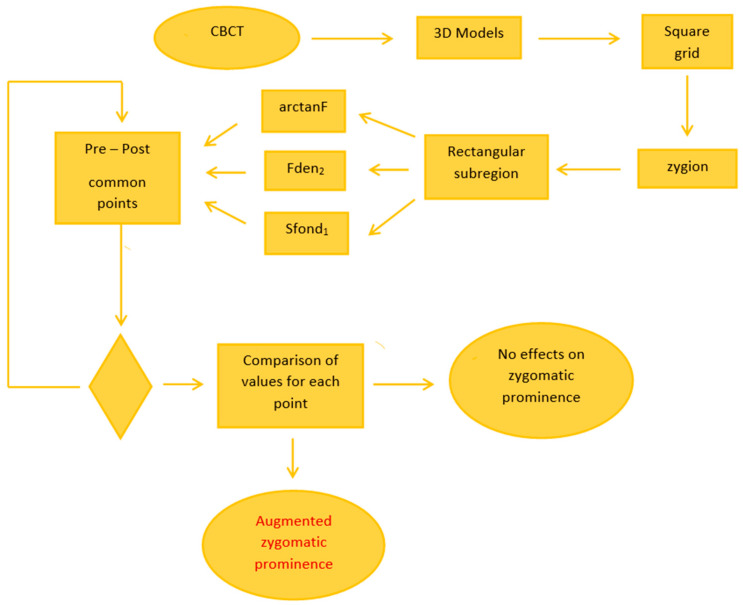
Overview of the experimental methodology; surgical stage is not included (CBCT: cone beam computed tomography).

The present study involved five patients (three females and two males) who underwent a single-stage bilateral malar valgization osteotomy in conjunction with maxilla-mandibular orthognathic procedures for functional and aesthetic correction, and three patients (one female and two males) who underwent the same surgeries except for the malar valgization osteotomy; this second group was considered as control group, as they did not undergo the surgery under focus. All considered patients were treated at the Division of Maxillofacial Surgery, San Giovanni Battista Hospital, University of Turin. Cone beam computed tomography (CBCT) of pre- and post-operative of patients was acquired. The inclusion criteria were (1) maxillary hypoplasia with relative excess mandibular growth and negative overjet of at least 4 mm, (2) inadequate cheekbone projection, (3) completion of growth (age range 21–43), (4) follow up at least one year, and (5) complete clinical and radiological records. The zygomatic valgization osteotomy has been performed following a standard Le Fort I osteotomy, according to the most recent published technique [18]. Surgical procedures have been performed by the same surgeon. The exclusion criteria were continued growth and history of craniofacial fractures or syndrome. Figure 1 reports the general overview of the methodology.

The study was conducted with the approval of the ethic committee of Città della Salute e della Scienza Hospital, Turin. Written informed consent was obtained from all the patients.

### 2.1. Surgical Procedure

As stated previously, the surgery was performed in accordance to Mommaerts [18,31,32]. First, standard Le Fort I osteotomy was performed, with conventional maxilla fixation by four plates with proper medial positioning of the plates on the zygomatic maxillary buttress. The periosteum was elevated in superior direction, laterally. The dissection stopped a few millimeters short of the inferior orbital rim, leaving the orbital septum untouched. The dissection continued along the superolateral direction with the creation of a tunnel to the junction between the frontal process of the zygoma and the zygomatic arch. Subperiosteal dissection was completed with the creation of a second tunnel posteriorly to the zygomatic buttress. A vertical osteotomy of the anterior and posterior sinus wall with a reciprocal saw was performed reaching the groove beneath the inferior orbital rim. After this, an oblique horizontal osteotomy from the temporo-frontal process transition zone was performed connecting the superior end of the vertical osteotomy, resulting in an open L-shaped region. A wide obwegeser osteotome was rotated outward and downward in the bone cuts to cause a greenstick fracture at the temporo-zygomatic suture; this way, the mobilization of the zygoma was achieved. The malar bone was pushed outward; this allowed a slight anterior movement of the zygoma. Finally, the zygoma was fixed with four screws in the desired position [31].

### 2.2. Data Processing and Geometrical Features Computation

**Figure 2:**
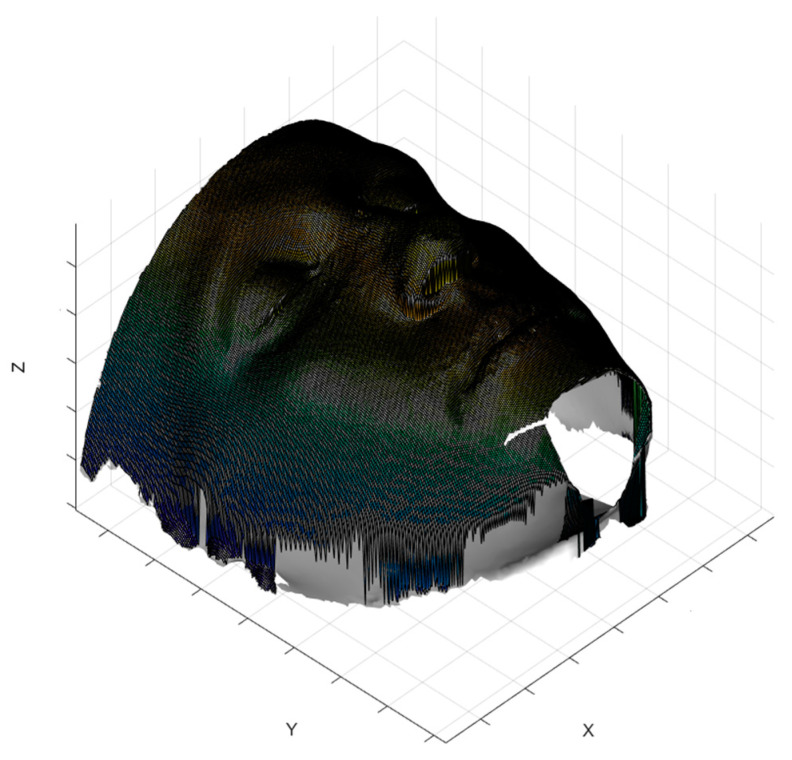
Example of the grid square surface computed from the triangular mesh.

**Figure 3:**
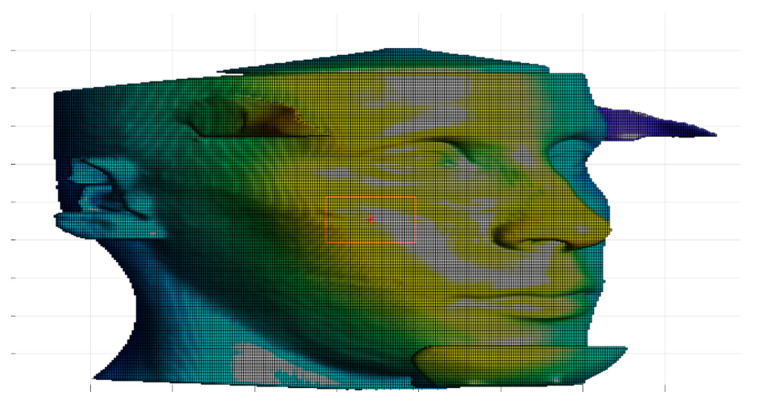
Rectangular facial subregion centered in the zygion.

After the collection of CBCT data, DICOM (digital imaging and communication in medicine) images were imported in Matlab^®^ (MathWorks Inc., Natick, MA, USA) the segmentation of the soft tissue was performed using k-means algorithm, and a further cleaning of images was carried on in order to avoid the inclusion of artifacts in the segmented images. To avoid an excessive loss of information when importing stl files generated with other software into Matlab^®^ environment, 3D models were generated through a Matlab^®^ implementation of marching cubes algorithm [33,34]; the triangular meshes were saved as stl files. For the computation of geometrical descriptors, the triangular meshes were turned into square grids and data were considered in the form of depth maps; more in detail, a Matlab^®^ algorithm was used to resample the triangular mesh in a square grid. The grid size (300 × 300) allowed us to obtain a sufficiently detailed surface. In Figure 2, an example of square grid surface overlying the triangular mesh is shown. To properly focus the analysis, the soft tissue zygion cephalometric landmark was chosen as reference point to identify a rectangular subregion of the face (Figure 3), on which geometrical descriptors were computed. To facilitate the correct positioning of the zygion, the 3D models were rotated 45 degrees to the left and right.

The zygion has been manually located on the pre- and post-operative depth maps of each subject; in order to avoid variance due to different positioning, it was located by the same operator. The geometrical descriptors here proposed are three among the derived and composed forms of primary descriptors that have already been presented in previous works by Marcolin and Vezzetti [29,30] where a detailed mathematical presentation is provided. The descriptors Fden_2_ and Sfond_1_ are composed descriptors derived from the primary descriptors F (coefficient of the first fundamental form) and S (shape index), respectively; this means that they are obtained from the combination of primary descriptors throughout standard mathematics. The descriptor arctanF is defined as a derived descriptor, as it is obtained through the application of standard arc tangent function to the primary descriptor F. A previous analysis [30] showed that Fden_2_, Sfond_1_, and arctanF descriptors highlight the contours of the face, leading to a more accurate discrimination of facial areas. Particularly, these descriptors assume values in the neighborhood of zero in correspondence of critical points, i.e., points on the dividing line between facial areas. The assumed values change sign accordingly to the derivatives with respect to the horizontal and vertical axes. Based on their behavior, these descriptors have been chosen as the most suitable for studying the variations in the shape of the face, in particular of the malar region.

**Figure 4:**
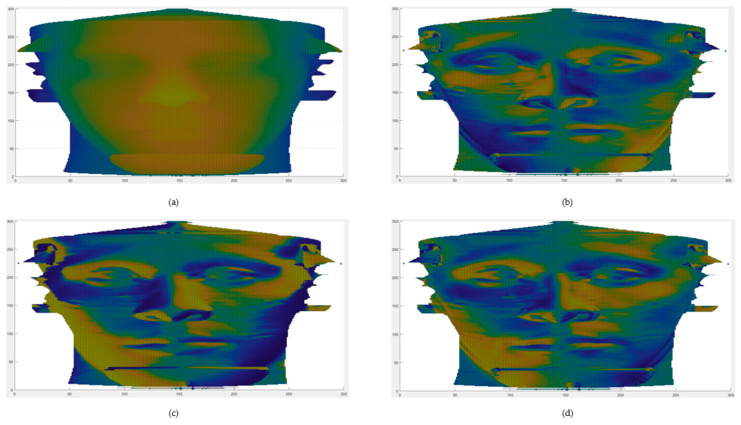
(**a**) Depth map of the post-operative of one of the involved patients. (**b**) Sfond_1_ computed on the same depth map. (**c**) arctanF computed on the same depth map. (**d**) Fden_2_ computed on the same depth map.

Differential geometry descriptors have shown their versatility and applicability in different fields, recently being applied in very different contexts: the first one related to face analysis applications [35], the second in the field of protein biophysics [36]. In Figure 4, the original facial surface and the three considered geometrical descriptors are shown. For the sake of representation, the total frontal facial surface is shown, even if the analysis was conducted on the rectangular subregion centered in the zygion point.

**Figure 5:**
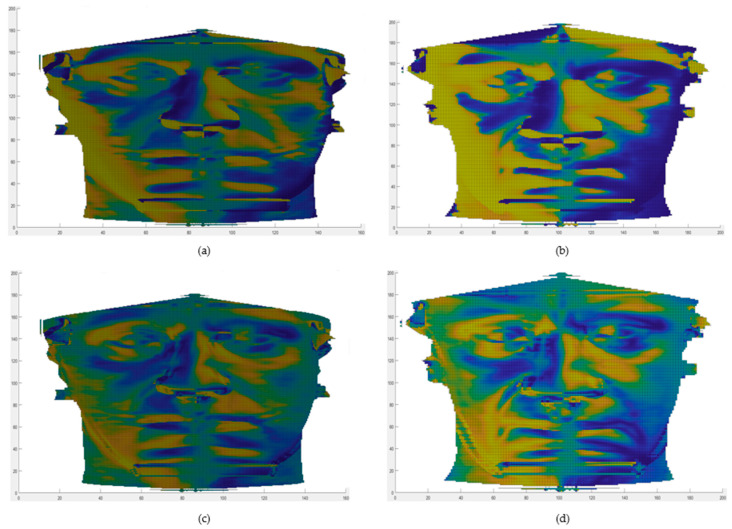
arctanF and Fden_2_ mapped on the pre- and on the post-operative depth maps of a patient underwent zygomatic valgization. In detail, (**a**) arctanF mapped on the pre-operative facial depth map, (**b**) arctanF mapped on the post-operative facial depth map, (**c**) Fden_2_ mapped on the pre-operative facial depth map, and (**d**) Fden_2_ mapped on the post-operative facial depth map.

**Figure 6:**
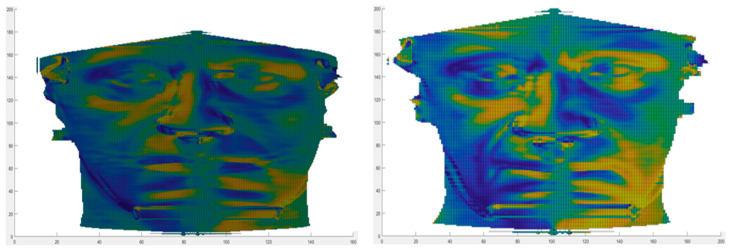
Sfond_1_ mapped on the pre- (**left**) and on the post-operative (**right**) depth maps of a patient underwent zygomatic valgization.

After the localization and the extraction of the rectangular region around the zygion, geometrical descriptors were calculated. The same process was performed on patients who underwent the malar valgization osteotomy and on the patients who did not. On both groups, each pair of pre- and post-operative rectangular region was superimposed, and only common points were considered (i.e., points that correspond on the pre- and on the post-operative surfaces). Right and left sides of the face were considered separately. For each of these points, the value of geometrical descriptors assumed in the pre-operative was compared with the value the same point assumed in the post-operative. Referring to the definitions of the primary geometrical descriptors F and S [29], an increase in the local values is related to a change in the shape of the surface. Therefore, the number of points for which the post-operative value was greater than the pre-operative value was computed. Due to the mathematical definitions of arctanF, Fden_2_, and Sfond_1_, the absolute values were considered. In Figure 5 and Figure 6, the geometrical descriptors computed on the pre- and on the post-operative of a patient underwent the zygomatic valgization are shown. Both figures refer to the same patient; as previously, to have a more effective representation, complete frontal depth maps are shown, even if the analyses were conducted on the rectangular zygion-centered subregion.

As stated before, the same geometrical descriptors were computed with the same modality on the left and right facial rectangular regions for patients who underwent maxilla-mandibular orthognathic procedures but not the malar augmentation. As previously, common points were compared between the pre- and the post-operative and the increasing or decreasing/stable values were collected.

**Figure 7:**
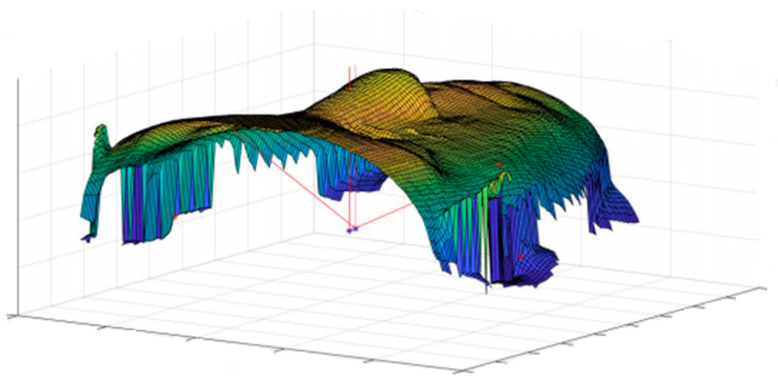
P_left_ and P_right_ located accordingly to the coordinates of nasion, zygion, and tragion.

To assess the malar variation, the angle describing the openness of the zygoma was considered as the ground truth. Its definition partially relied on the one described by Lehre et al. [16]. In their study, the angle was defined on the hard tissue, while here it was defined between soft tissue cephalometric landmarks. Moreover, the angle considered by Lehre et al. was the one defined by the symmetry axis of the cranium and the straight line linking the clivus and the apex of the zygoma. This way, facial asymmetry was not considered. Instead, in the current study the considered angle α was constructed between the symmetry axis of the face; the point P defined by the coordinates of nasion, zygion, and tragion; and the zygion. As for the zygion, the nasion and the tragion were located manually by the same operator. To overcome the possible presence of facial asymmetry, right and left sides of the facial surface were considered separately. This led to the identification of two points P, namely, P_right_ and P_left_, located accordingly to the coordinates of right and left nasion, right and left zygion and right and left tragion. Specifically, the generic P point is located at the x-coordinate of the nasion, at the y-coordinate of the zygion and at the z-coordinate of the tragion (Figure 7).

## 3. Results

In Table 1, the number of points for which the arctanF value increased and for which it decreased or remained stable for the patients underwent malar valgization is reported.

For all patients who underwent the malar augmentation, arctanF highlighted a change in the facial shape; particularly, in all cases more than 55% of the points showed values of arctanF that have risen from pre- to post-operative. In details, for patient 1 the number of points for which arctanF values risen from pre- to post-operative was the 60% and the 78% of the total number, for right and left, respectively. For patient 2, the percentages were 59% for the right subregion and 56% for the left one. Patient 3 showed higher values for both right and left, with 71% and 76%, respectively. Similar to patient 1 and patient 2, in patient 4 the right side gave a result of 59%, while on the left side the total number of considered points increased its value (100%). Finally, for patient 5 it resulted a percentage of 77% on the right side and of 74% on the left side.

As for arctanF, Fden_2_ highlighted the modification of the facial shape in patients with malar augmentation. The obtained results were similar to the ones obtained from the computation of arctanF; it was to be expected as the primary geometrical descriptor is the same for both (Table 2). The results were, in this case, right and left side, respectively: 60% and 78% for patient 1, 60% and 78% for patient 2, 72% and 75% for patient 3, 59% and 100% for patient 4, and 77% and 73% for patient 5.

The last geometrical descriptor for which data were collected, Sfond_1_, showed for the patients underwent valgization a trend similar to the ones previously described. As for arctanF and Fden_2_, the total number of patients registered an increasing in the value of this feature, highlighting that a change in the shape of the malar region occurred. In Table 3, the results for Sfond_1_ are collected.

For patient 1, the right side showed an increasing in the 60% of the total number of points; on the left side the increasing involved 78% of the totality. Patient 2 showed a result similar to that of patient 1 for the right side (59%) and a lower percentage for the left (56%). As for arctanF and Fden_2_, the results obtained for patient 3 are higher than the first two subjects; the right side increased in 72% of cases, and the left one in 76%. As previous, patient 4 showed a strong asymmetry in the facial modification; in fact, Sfond_1_ values increased on the right malar subarea in 59% of the points, while the percentage reaches 100% on the left one. This behavior suggested that a strong facial asymmetry characterized patient 4. Patient 5 showed the higher value concerning the right side (77%) and a slightly lower result for the left side (72%).

To have a counterproof of the effectiveness of the three geometrical descriptors involved in this study, the same features were computed under the same conditions on three patients that have not been treated for flat cheekbones. Consequently, they underwent only maxilla-mandibular orthognathic surgeries.

The results derived from the analysis of arctanF are reported in Table 4. The behavior of the points in the rectangular region differed significantly from the patients who underwent malar augmentation; here, the majority of the points showed a value of arctanF stable from pre- to post-operative or higher in the pre- than in the post-operative. More in detail, for patient 1 only 23% and 9% of points increased its arctanF value after the surgeries, right and left, respectively. For patient 2, the totality of the points both for right and left did not show an increasing in the post-operative. Patient 3 showed similar results, with 25% of points with increased values on right side and 0% on left side.

Fden_2_ highlighted a behavior very similar to the one resulting from arctanF, with percentages of increased values lower than 24% (Table 5). The same results were obtained with Sfond_1_ (Table 6).

From the comparison of the results obtained for the five patients who underwent malar augmentation and for the three patients who did not, it was possible to assert that a change occurred in the shape of the first group. Particularly, as the control group and the test group underwent the same maxilla-mandibular orthognathic surgeries, it could be supposed that the modifications highlighted by the geometrical descriptors in the test group were associated to the valgization osteotomy. This suggested that the three proposed geometrical features were very sensible to the modification in the surface due to this type of surgical intervention.

As described in the previous section, to assess the changes due to the zygoma valgization, the angle quantifying the zygoma openness was considered. This feature was considered as ground truth for the evaluation of malar augmentation as it seemed less influenced by the other performed surgeries compared with other features like volume and area. In Table 7, the values of the angle α for test group are reported. Except for patient 2, there was an increase in the opening of the zygoma, both right and left. For patient 1, the increase is significantly more pronounced on the right angle (31%) than the left one (21%). Patient 3 showed percentage variations more similar on right and left, with 11% and 10%, respectively. Patient 4 showed an increasing of zygoma openness of 16% on right side and 14% on left side. The last patient of test group, patient 5, reported an increasing in the openness of right angle α of 30%, while a dramatically lower percentage is reported for left angle α (8%). The paired sample Wilcoxon test was performed with a significance level of 0.05; the resulting *p*-value was lower than the significance level both for right and left zygoma, showing a statistically significant difference between the pre- and the post-operative measurements. Particularly, it resulted that the pre-operative values were significantly lower than the post-operative ones.

Table 8 reports the angle values obtained for the control group. Paired sample Wilcoxon test was performed, with a significance level set to 0.05. From this analysis, it resulted that no statistically significant differences occurred between pre- and post-operative measurements (*p* > 0.05).

## 4. Discussion

Maxillo-mandibular orthognathic surgeries such as Le Fort I osteotomy and bilateral sagittal split osteotomy (BSSO) are commonly performed on patients with severe dentofacial deformities. These surgeries address malocclusion and unaesthetic of the lower part of the face, while they do not specifically address the unaesthetic of flatness of the malar region, that often is associated with maxillary deficiency and excessive relative mandibular growth. Thus, zygomatic valgization osteotomy can be performed to enhance the fullness of the zygoma, leading to a more satisfactory aesthetical outcome. Several studies have been carried on in order to evaluate the changes occurring in the malar region after this type of intervention, particularly considering the volume and the openness of the zygoma [37].

The aim of this study was to propose a novel approach to the analysis of facial modifications after surgeries by means of three-dimensional geometrical descriptors usually addressed to face analysis purposes, such as face recognition (FR) [26] and facial expression recognition (FER) [28]. In the context of facial analysis, several descriptors have been proposed in the 3D domain. Principal component analysis-based model (PCA) [38,39,40], region-based deformable model (R3DM) [41], and closest normal points (CNPs) [42] are just some examples among the holistic methods, in which the entire face model is required. On the other hand, the feature-based methods seem to be more suitable as their application is possible even without a complete facial model [43]. Geometric and local shape descriptors seem to be promising in face recognition systems that are robust to distortions caused by facial expressions. Abbad et al. [44] proposed an adaptation of the wave kernel signature (WKS) for shape analysis of the face as a local descriptor to construct a hybrid method involving radial and level curves of the face and the local information in the WKS. Local binary pattern (LBP) showed good results in 2D face analysis, and it was adapted to extract texture features on 3D facial depth images; the so-extracted features were suitable to fed a support vector machine algorithm (SVM) for classification [45]. Among the geometrical descriptors proposed in literature, arctanF, Fden_2_, and Sfond_1_ were judged the most effective to highlight shape modifications of the malar region.

Despite the broad panorama of descriptors for facial analysis, to our knowledge none of these have ever been used to assess changes in the facial shape of orthognathic patients, making it difficult to compare the results obtained in this study with other descriptors; for this reason, an angular measure already described in a previous work [37] was considered as ground truth. Despite the small size of the sample of patients for both the test and the control group, the results seem to be promising; the adopted geometrical descriptors showed a high response in the detection of the changes occurred in the facial shape for the malar augmentation group (in all cases more than 55% of the considered points showed an increasing of the values of these features). On the other hand, geometrical descriptors did not highlight a significant variation in the shape of the zygoma for the control group. This behavior was expected according to the performed surgeries. Moreover, the selection of a small rectangular area centered in the zygion helped in focusing only on the soft tissue modifications due to zygomatic valgization rather than to the conjunction of maxillo-mandibular orthognathic surgeries. The value of this 3D geometrical approach seems to be strengthened by the fact that it outperformed the angular measure that was considered to assess the modification of the zygoma. In fact, from the comparison of the results, it is visible that geometrical descriptors have been able to detect changes in malar shape in all the patients in the sample, while the angle α gave conflicting values for patient 2. Particularly, unless patient 2 presented values suggesting a less visible modification, arctanF, Fden_2_, and Sfond_1_ were able to detect it, contrary to α. Moreover, by definition the angle α is strongly influenced by the identification of the zygion position, so by the experience and the precision of the operator. An advantage of the approach with geometrical descriptors is that the imprecision that might occur in locating the zygion is balanced by (1) the rectangular region constructed around the landmark and (2) the fact that the ensemble of values resulting from the computation of the geometrical descriptors gives the measure of a change of shape, which is not related to the operator’s accuracy.

Basing on these encouraging results, the following steps would be (1) the boost of the sample size, for both test and control group; (2) the quantification of the shape variation; and (3) the correlation of zygomatic soft tissue shape variation with bony movements; this way, a deeper knowledge of the modifications of malar region would be accessible and it could be involved in the development of predictive surgical tools.

## 5. Conclusions

The results obtained from this study show the suitability of the geometrical descriptors in fields other than FR and FER; in fact, their high sensibility to detect and describe changes occurring in patients who underwent malar augmentation make them a valuable tool in the field of maxillofacial surgery. This conclusion is drawn from the consideration that, even on a small sample, these features were able to detect the increasing in the prominence of the zygoma in the patients of the zygomatic valgization group, while no significant modifications were detected on the patients of the control group.

## Figures and Tables

**Table 1:** Patients underwent zygomatic valgization osteotomy. The count of the points for which the value of arctanF increased (third column) and for which the value of arctanF decreased or remained unchanged (fourth column) is reported.

arctanF		^1^ # Post-Operative Increased Values	^1^ # Post-Operative Decreased/Unchanged Values
Patient 1	right	133	91
	left	450	126
Patient 2	right	476	334
	left	374	298
Patient 3	right	214	86
	left	539	174
Patient 4	right	120	84
	left	100	0
Patient 5	right	214	65
	left	242	87

^1^ # indicates the number of points.

**Table 2:** Patients underwent zygomatic valgization osteotomy. The count of the points for which the value of Fden_2_ increased (third column) and for which the value of Fden_2_ decreased or remained unchanged (fourth column) is reported.

Fden_2_		^1^ # Post-Operative Increased Values	^1^ # Post-Operative Decreased/Unchanged Values
Patient 1	right	134	90
	left	451	125
Patient 2	right	487	323
	left	451	125
Patient 3	right	215	85
	left	538	175
Patient 4	right	120	84
	left	100	0
Patient 5	right	216	63
	left	234	85

^1^ # indicates the number of points.

**Table 3:** Patients underwent zygomatic valgization osteotomy. The count of the points for which the value of Sfond_1_ increased (third column) and for which the value of Sfond_1_ decreased or remained unchanged (fourth column) is reported.

Sfond_1_		^1^ # Post-Operative Increased Values	^1^ # Post-Operative Decreased/Unchanged Values
Patient 1	right	135	89
	left	452	124
Patient 2	right	480	330
	left	373	299
Patient 3	right	215	85
	left	539	174
Patient 4	right	121	83
	left	100	0
Patient 5	right	215	64
	left	231	88

^1^ # indicates the number of points.

**Table 4:** Patients who had not malar augmentation. The count of the points for which the value of arctanF increased (third column) and for which the value of arctanF decreased or remained unchanged (fourth column) is reported.

arctanF		^1^ # Post-Operative Increased Values	^1^ # Post-Operative Decreased/Unchanged Values
Patient 1	right	103	345
	left	27	259
Patient 2	right	0	372
	left	0	253
Patient 3	right	36	107
	left	0	186

^1^ # indicates the number of points.

**Table 5:** Patients who had not malar augmentation. The count of the points for which the value of Fden_2_ increased (third column) and for which the value of Fden_2_ decreased or remained unchanged (fourth column) is reported.

Fden_2_		^1^ # Post-Operative Increased Values	^1^ # Post-Operative Decreased/Unchanged Values
Patient 1	right	103	345
	left	26	260
Patient 2	right	0	372
	left	0	253
Patient 3	right	37	106
	left	0	186

^1^ # indicates the number of points.

**Table 6:** Patients who had not malar augmentation. The count of the points for which the value of Sfond_1_ increased (third column) and for which the value of Sfond_1_ decreased or remained unchanged (fourth column) is reported.

Sfond_1_		^1^ # Post-Operative Increased Values	^1^ # Post-Operative Decreased/Unchanged Values
Patient 1	right	103	345
	left	26	260
Patient 2	right	0	372
	left	0	253
Patient 3	right	37	106
	left	0	186

^1^ # indicates the number of points.

**Table 7:** Angle α for zygomatic valgization group (test group).

	αR Pre-op ^1^	αR Post-op ^1^	αL Pre-op ^1^	αR Post-op ^1^
Patient 1	39.93	52.37	37.99	45.97
Patient 2	49.34	38.26	50.09	42.11
Patient 3	40.77	45.15	43.61	47.95
Patient 4	43.16	50.24	44.09	50.17
Patient 5	48.83	63.27	58.72	63.51

^1^ R = right; L = left; pre-op = pre-operative; post-op = post-operative.

**Table 8:** Angle α for control group.

	αR Pre-op	αR Post-op	αL Pre-op	αR Post-op
Patient 1	45.19	42.22	41.75	42.36
Patient 2	47.24	31.34	42.50	37.12
Patient 3	47.31	48.01	47.50	46.27

## Data Availability

No new data were created in this study. Data sharing is not applicable to this article.
